# Carboxymethylation of *Desmodium styracifolium* Polysaccharide and Its Repair Effect on Damaged HK-2 Cells

**DOI:** 10.1155/2022/2082263

**Published:** 2022-08-12

**Authors:** Gu-Hua Tang, Jing-Hong Liu, Xin-Yuan Sun, Jian-Ming Ouyang

**Affiliations:** ^1^College of Chemistry and Materials Science, Institute of Biomineralization and Lithiasis Research, Jinan University, Guangzhou 510632, China; ^2^Department of Urology, Guangzhou Institute of Urology, Guangdong Key Laboratory of Urology, The First Affiliated Hospital of Guangzhou Medical University, Guangzhou Medical University, Guangzhou, Guangdong 510230, China

## Abstract

**Objective:**

*Desmodium styracifolium* is the best traditional medicine for treating kidney calculi in China. This study is aimed at increasing the carboxyl (-COOH) content of *D. styracifolium* polysaccharide (DSP0) and further increasing its antistone activity.

**Methods:**

DSP0 was carboxymethylated with chloroacetic acid at varying degrees. Then, oxalate-damaged HK-2 cells were repaired with modified polysaccharide, and the changes in biochemical indices before and after repair were detected.

**Results:**

Three modified polysaccharides with 7.45% (CDSP1), 12.2% (CDSP2), and 17.7% (CDSP3) -COOH are obtained. Compared with DSP0 (-COOH content = 1.17%), CDSPs have stronger antioxidant activity in vitro and can improve the vitality of damaged HK-2 cells. CDSPs repair the cell morphology and cytoskeleton, increase the cell healing ability, reduce reactive oxygen species and nitric oxide levels, increase mitochondrial membrane potential, limit autophagy level to a low level, reduce the eversion of phosphatidylserine in the cell membrane, weaken the inhibition of oxalate on DNA synthesis, restore cell cycle to normal state, promote cell proliferation, and reduce apoptosis/necrosis.

**Conclusion:**

The carboxymethylation modification of DSP0 can improve its antioxidant activity and enhance its ability to repair damaged HK-2 cells. Among them, CDSP2 with medium -COOH content has the highest activity of repairing cells, whereas CDSP3 with the highest -COOH content has the highest antioxidant activity. This difference may be related to the active environment of polysaccharide and conformation of the polysaccharide and cell signal pathway. This result suggests that *Desmodium styracifolium* polysaccharide with increased -COOH content may have improved potential treatment and prevention of kidney calculi.

## 1. Introduction

Kidney calculi is a common clinical disease, and its incidence has increased significantly in the past 30 years [1]. However, given the incomplete elucidation of its mechanism, effective clinical treatment drugs and preventive measures remain lacking. Most patients need to be treated many times in their lifetime because of repeated attacks of stones, thereby bringing heavy burdens to individuals, families, and society.

Evidences showed that calcium oxalate (CaOx) kidney calculi is closely related to the damage of renal epithelial cells [2]. For example, the fragments released after the damage of renal epithelial cells can promote the growth of CaOx crystals and promote their adhesion onto the cell surface by providing heterogeneous nucleating substances, thereby increasing the risk of stone formation [3, 4].

Studies showed that the exposure of high concentration of oxalate to renal epithelial cells increases the production of free radicals [5] and reduces cell viability and mitochondrial membrane potential [6], thereby leading to cell oxidative damage. Liang et al. [7] found that the androgen receptor (AR) signal carried by men with high frequency of kidney calculi can increase oxalate biosynthesis and oxidative stress and cause damage to renal tubular epithelial cells. When repaired, injured cells can reduce the adsorption of Ca^2+^ in urine [8] and the adhesion to urine crystallites [9], thus inhibiting the formation of CaOx crystals.

Plant polysaccharides have antioxidant, antitumor, antiviral, and immunomodulatory activities [10–13]. However, natural polysaccharides usually have limited clinical applications due to their high molecular weight and low active group content. The chemical modification of polysaccharides, such as sulfation and carboxymethylation, to increase the content of active groups of polysaccharides can improve the biological activity of polysaccharides. For example, the carboxymethylation of polysaccharides is conducive to improve the water solubility [14] and antioxidant activity [15] of polysaccharides. Our previous study also showed that the three carboxymethylated *Poria cocos* polysaccharides obtained by modification exhibit excellent antioxidant activities, and their abilities of scavenging free radicals (•OH and DPPH) and chelating Fe^2+^ are positively correlated with the degree of carboxymethylation [15]. Evidence shows that carboxyl (-COOH) plays a major role in the antitumor activity of *Ganoderma applanatum* polysaccharide [16]. Li et al. [17] showed that the carboxymethylation of *Morchella angusticepes* polysaccharide improves its cholesterol-lowering activity in rats. Carboxymethylated corn bran polysaccharide shows significant antitumor activity in vitro against human lung (A549) and human liver (HepG-2) cancer cells [18]. Deng et al. [19] performed the chemical modification on *Mori Fructus* polysaccharide (MFP) and indicated that the carboxymethylated derivative (C-MFP) exhibits a more significant hepatoprotective effect than the sulfated and oxidative degradation derivatives, indicating that C-MFP can be used to reduce liver injury.

Carboxymethylation can enhance the antioxidant activity of polysaccharides related to the following factors: first, the introduction of the substituent group -CH_2_COOH changed the configuration of the polysaccharide, weakened the hydrogen bond dissociation energy in the polysaccharide molecule, and improved the hydrogen supply capacity of the polysaccharide [20]. Second, carboxymethylation can improve the water solubility of polysaccharides, increase the degree of freedom of polysaccharides, and enhance their activity [21, 22]. Third, as the content of -COOH in carboxymethylated polysaccharides increased, the ability to provide single electrons or hydrogen atoms increased, and thus, the ability to scavenge free radicals increased, so the antioxidant activity increased [15]. In addition, with the increase of the content of -COOH in polysaccharides, the ability of chelating transition metal ions (such as Fe^2+^ and Cu^2+^) is enhanced, thus inhibiting the production of free radicals. Fourth, carboxymethylation can expand the structure of polysaccharide and enhance its biological activity. Some literatures attributed the enhancement of the activity of polysaccharides after carboxymethylation to the conformational expansion of polysaccharides caused by the presence of -COOH, which interacted with some protein on the cell surface [10, 23, 24]. Chen et al. [24] reported that the relative extended chain conformation of carboxymethylated derivatives is beneficial to enhance antitumor activity by interacting with macrophage surface receptors to activate immune function.

*Desmodium styracifolium* (DS) is the best traditional Chinese medicine for the treatment of kidney calculi [25]. The biological activity of polysaccharide is affected by its structure (molecular weight (*Mw*), monosaccharide composition, hydroxyl content, glycosidic bond type, degree of branching, change of higher stereostructure, and chain conformation) [26, 27]. *Desmodium styracifolium* polysaccharide (DSP) is composed of mannose, rhamnose, galacturonic acid, glucose, galactose, xylose, and arabinose. The research by Zhang et al. [28] showed that glucose and mannose had a more significant effect on the free radical scavenging capacity. Gu et al. [29] reported that low *Mw* polysaccharides from thirteen boletus mushrooms have strong antioxidant activity, because low *Mw* polysaccharides can present higher content of reducing end groups and accept more free radicals. As carboxyl groups in uronic acid may act as hydrogen donors and electron transfer agents [30], the increase of acidic groups in polysaccharides is beneficial to their antioxidant activity [31]. The water solubility of low molecular weight polysaccharides is often increased, so that they will have higher degree of freedom [32] and smaller steric hindrance, which are all conducive to improving their antioxidant activity [33]. Xiang et al. [34] extracted DS polysaccharide (DSP) by *n*-butyl alcohol and observed reduced deposition of CaOx crystals in kidney and prevented the change in renal toxicity and inhibition of urolithiasis.

To improve the antikidney calculi activity of the initial DSP (DSP0), we modified DSP0 with 1.17% carboxyl (-COOH) content by carboxymethylation at varying degrees and obtained three modified polysaccharides with -COOH contents of 7.45% (CDSP1), 12.2% (CDSP2), and 17.7% (CDSP3). The differences in antioxidant activity and the ability to repair damaged HK-2 cells of these polysaccharides were compared. This study is expected to provide enlightenment for the development of new antistone drugs.

## 2. Experiments and Methods

### 2.1. Materials and Apparatus

#### 2.1.1. Materials

*Desmodium styracifolium* polysaccharide (DSP) was provided by Xi'an Qingzhi Biotechnology Co., Ltd., with the polysaccharide content of 95%, and the other 5% being a very small amount of impurities such as phenols, flavonoids, protein, pigment, and salt. DSP0 was obtained from *Desmodium styracifolium* by hot water extraction.

DSP0 was purified by the following methods: (1) dissolve DSP0 in distilled water, and remove protein by using the Sevag method; (2) dialyze with a 3000 Da dialysis membrane for 3 days, and evaporate the dialyzed polysaccharide solution under reduced pressure; (3) precipitate with anhydrous ethanol for 12 h, and the obtained precipitate was collected by centrifugation and dried at 60°C. Phenols, flavonoids, and other compounds have been basically removed in this series of purification processes [35]. We used the UV method to scan the polysaccharide aqueous solution in the range of 200-300 nm, and no absorption peak was found in this range [36]. The ninhydrin experiment showed no purple, which indicated that the polysaccharide no longer contained protein component [37]. Total phenolic compounds were determined throughout the Folin–Ciocalteu method [38], and no phenols were found.

Conventional chemical reagents such as chloroacetic acid and oxalate were analytical reagents (Guangzhou Chemical Reagent Factory, China), phosphate-buffered saline (PBS, Gbico, pH = 7.4). The experimental water was secondary distilled water.

Human kidney proximal tubular epithelial (HK-2, Shanghai Cell Bank, Chinese Academy of Sciences), fetal bovine serum (FBS) and Dulbecco's modified Eagle's medium (DMEM/F-12) were all obtained from Gbico. 4′,6-Diamidino-2-phenylindole (DAPI) staining solution, hematoxylin-eosin (HE) kit, active oxygen detection kit (DCFH-DA), mitochondrial membrane potential assay kit (JC-1), calcein/PI cell viability, Hoechst 33342 kit are all made by Shanghai Beyotime Biotechnology Co., Ltd. (Shanghai, China); Annexin V–FITC/PI Apoptosis Kit (BD Company, USA); autophagy detection kit (MDC, Solarbio Life Sciences Co., Ltd.); serum albumin (BSA) and Actin-Tracker Green (Jiangsu KeyGen Biotechnology Co., Ltd., Nanjing, China).

#### 2.1.2. Apparatus

The apparatus are as follows: Equinox55 Fourier transform infrared absorption spectrometer (Germany); magnetic resonance apparatus (Varian Bruker-600mhz); laser confocal microscope (LSM 880 with AiryScan, Carl Zeiss, Germany); inverted fluorescence microscope (Leica DMRA2, Germany); microplate reader (Gen5, BioTek, the US); flow cytometry (FACS Canto, BD, the US); optical microscopy (OLYMPUS, CKX41, Japan); Thermo ICS5000 ion chromatographic system (ICS5000, Thermo Fisher Scientific, USA).

### 2.2. Experimental Methods

#### 2.2.1. Carboxymethylation of Polysaccharides

According to reference [39], DSP0 polysaccharide (250 mg) was suspended in 10 mL isopropanol at room temperature and stirred for 15 mins. Then, 15 mL of 30% NaOH solution (*w*/*v*) was slowly added to the mixture and stirred at 50°C until the polysaccharide was completely dissolved. Then, while stirring, 1 g, 2 g, and 3 g chloroacetic acid (suspended in 3 mL distilled water) were added, reacted at 50°C for 8 h, then cooled to room temperature and neutralized with 1.0 M HCl. The resulting solution was dialyzed with a 3000 Da dialysis membrane for 3 days, and the dialyzed products were evaporated under reduced pressure, concentrated, and centrifuged to obtain modified polysaccharides CDSPs with different -COOH contents (Table 1).

#### 2.2.2. Characterization of Polysaccharides

*(1) Determination of Molecular Weight*. According to the method in literature [40], the molecular weight of polysaccharides was determined by Ubbelohde viscosity method at 25 ± 0.2°C, and the average value of three times was taken. The intrinsic viscosity [*η*] and molecular weight *M* of DSPs can be expressed by the Mark-Houwink empirical equation: [*η*] = *κ* *M*^*α*^. For DSPs, the parameters in the equation are *κ* = 2.0 × 10^−4^, and *α* = 1.1.

**Figure 1:**
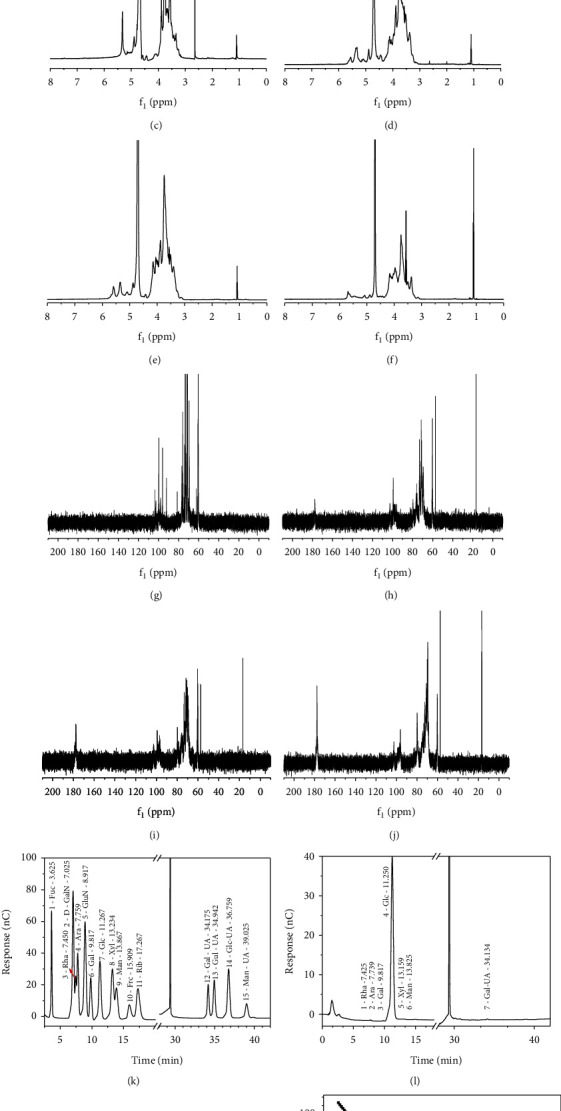
Characterization of CDSPs with different –COO^−^ contents. (a) FT-IR spectrum; (b) a plot of the intensity of COOH and its related absorption peaks versus the content of –COOH in the polysaccharide; (c) ^1^H NMR of DSP0; (d) ^1^H NMR of CDSP1; (e) ^1^H NMR of CDSP2; (f) ^1^H NMR of CDSP3; (g) ^13^C NMR diagram of DSP0; (h) ^13^C NMR of CDSP1; (i) ^13^C NMR of CDSP2; (j) ^13^C NMR of CDSP3; (k) standard sample ion chromatogram; (l) ion chromatogram of DSP0; (m) parameters for the determination of -COOH content in CDSP2 by conductivity titration; (n) comparison of titration curves for the determination of -COOH content in four polysaccharides.

*(2) Determination of -COOH Content*. The conductivity titration method was used to determine the content of -COOH in polysaccharides [41]. Take the conductivity as the ordinate and the consumed NaOH volume as the abscissa to draw the conductivity titration curve. The titration curve of conductivity can be divided into three sections, namely, a significant decline stage (*A*), an equilibrium stage (*B*), and a significant increase stage (*C*); make three tangents for these three stages, respectively, and the intersection point is the stoichiometric point. Among them, the intersection of line *A* and line *B* gives the NaOH volume *V*_1_ required for excessive hydrochloric acid consumption, and the intersection of line *B* and line *C* gives the NaOH volume *V*_2_ consumed by excessive hydrochloric acid and -COOH in DSP, and *V*_2_ − *V*_1_ is the NaOH volume (platform part) consumed by -COOH in polysaccharide (Figure 1(m)). Therefore, the percentage of -COOH can be calculated according to the following formula:

(1) $$\begin{array}{c} -\text{COOH}\left(\%\right)=\frac{C_{\text{NaOH}}\times \left(V_{2}-V_{1}\right)\times 45/1000}{C_{\text{sample}}\times 40/1000}\times 100. \end{array}$$

In the equation, *C*_NaOH_ is the molar concentration of NaOH solution (mol/L), *C*_Sample_ is the concentration of polysaccharide (g/L), 45 (g/mol) is the molar mass of -COOH, and 40 (mL) is the volume of polysaccharide solution in the test. Each experiment was repeated 3 times and averaged.

*(3) Determination of Monosaccharide Composition*. According to reference [42], the monosaccharide components of DSP0 were analyzed and detected by electrochemical detector using Thermo ICS5000 ion chromatography system.

Sample preparation and extraction: approximately 5 mg of sample was hydrolyzed with trifluoroacetic acid (2 M) at 105°C for 6 h in a sealed tube. Dry the sample with nitrogen. Add methanol to wash, then blow dry, and repeat methanol wash 2–3 times. The residue was redissolved in deionized water and filtered through 0.22 *μ*m microporous filtering film for measurement.

HPAEC conditions: the sample extracts were analyzed by high-performance anion-exchange chromatography (HPAEC) on a CarboPac PA-10 anion-exchange column (4.6 by 250 mm; Dionex) using a pulsed amperometric detector (PAD; Dionex ICS 5000 system). Flow rate, 0.5 mL·min^−1^; injection volume, 5 *μ*L; solvent system: 0.1 M NaOH, 0.2 M NaAc; gradient program, 95 : 5 *V*/*V* at 0 min, 80 : 20 *V*/*V* at 30 min, 60 : 40 *V*/*V* at 30.1 min, 60 : 40 *V*/*V* at 45 min, 95 : 5 *V*/*V* at 45.1 min, 95 : 5 *V*/*V* at 60 min. The quantitative standard curve was established by using the standard products (fucose, rhamnose, arabinose, galactose, glucose, xylose, mannose, fructose, ribose, galacturonic acid, glucuronic acid, mannuronic acid, guluronic acid, D-galactosamine, and D-glucosamine) purchased from the Sigma Company.

*(4) FT-IR Characterization*. The completely dried polysaccharide sample 2.0 mg and KBr sample 200 mg were mixed, ground into powder, pressed, and scanned in the wave number range of 4000-400 cm^−1^.

*(5) ^1^H Nuclear Magnetic Resonance (NMR) and ^13^C NMR Characterization*. Refer to reference [43], weigh 20 mg of completely dried polysaccharide sample, dissolve it in a nuclear magnetic tube filled with 0.5 mL deuterated water (D_2_O), and detect it by nuclear magnetic resonance spectrometer. The scanning times of ^13^C NMR of DSP0, CDSP1, CDSP2, and CDSP3 are 949, 872, 2000, and 4009, respectively, and the scanning times of ^1^H NMR are 16, 16, 64, and 16, respectively. Scanning temperature: 298°C.

#### 2.2.3. In Vitro Antioxidant Activity of CDSPs

H_2_O_2_/Fe system [44] and absolute ethanol system [45] were used to detect the scavenging ability of polysaccharides on •OH and DPPH radicals in vitro, and vitamin C was used as positive control group. Each experiment was repeated three times, and the average value was taken.

#### 2.2.4. Detection of Cell Viability

*(1) Cell Culture*. HK-2 cells were cultured in DMEM-F12 medium containing 10% fetal bovine serum, 1% penicillin, and streptomycin in an incubator at 37°C, 5% CO_2_, and saturated humidity. Trypsin digestion was used for cell passage. In all experiments, the cells were treated after being cultured with seed plate for 24 h to reach 80% confluence.

*(2) Selection of Polysaccharide Concentration*. By setting four polysaccharide concentrations of 40, 60, 80, and 100 *μ*g·mL^−1^, CCK-8 method was used to detect the toxicity of polysaccharide to cells and cell viability, so as to find out the best repair concentration of CDSPs.

HK-2 cells were inoculated into 96-well plate at a density of 1 × 10^5^ mL^−1^ and 100 *μ*L each well and incubated in an incubator at 37°C and 5% CO_2_ for 24 h, then incubated in serum-free culture medium for 12 h, and the culture medium was discarded, and the cells were washed twice with PBS. The models were divided into three groups:

1. Normal control group (NC): serum-free medium was added
2. Oxalate damage control group (DC): HK-2 cells were injured by adding phosphate buffer saline (PBS) solution containing 2.8 mM oxalate for 3 h
3. Polysaccharide repair group: HK-2 cells were injured by adding PBS solution containing 2.8 mM oxalate for 3 h, and then different CDSPs with final concentrations of 40, 60, 80, and 100 *μ*g·mL^−1^ were added to repair the damaged HK-2 cells for 12 h

Each experiment was set up with 5 multiple wells, which were detected in strict accordance with CCK-8 kit, and the OD value was detected by microplate reader at the wavelength of 450 nm.

#### 2.2.5. Observation of HE Staining

The experimental grouping was the same as that in Section 2.2.4, and 80 *μ*g·mL^−1^ DSPs were selected for the repair of damaged HK-2 cells. After reaching the culture time, the supernatant was sucked off, fixed with 4% paraformaldehyde, stained with hematoxylin and eosin in sequence for a certain time, and washed with PBS several times. After observation under the microscope, the nucleus was blue-purple, while the cytoplasm was pink or red.

#### 2.2.6. Observation of Cytoskeleton

The experimental grouping was the same as that in Section 2.2.5. At the end of the culture time, the cells were fixed with 4% paraformaldehyde solution for 20 mins, washed with 0.1% Triton X-100 PBS, diluted with 1–5% BSA and 0.1% Triton X-100 PBS in the ratio of 1 : 100, incubated at room temperature in the dark for 20–60 mins, and then, observed under confocal microscope.

After the incubation time, the cells were fixed in 4% paraformaldehyde solution for 20 mins, washed fully with 0.1% Triton X-100 PBS, diluted with 1–5% BSA and 0.1% Triton X-100 PBS in a 1 : 100 ratio in Actin-tracker Green staining working solution, and incubated in the dark for 20–60 mins at room temperature. Afterwards, the cells were observed under a laser confocal microscope.

#### 2.2.7. Detection of Cell Healing Ability

The experimental grouping was the same as that in Section 2.2.5. After reaching the culture time, the supernatant was sucked off, and a sterile 200 *μ*L gun head was used to mark the sample petri dish in a certain direction. After washed twice with PBS, fresh medium was added for continuous culture, and the changes of scratch spacing were observed under a light microscope at regular intervals, photographed, and retained, and the cell healing rate was calculated. In the experiment, marker were used to mark lines at the bottom of the well plates in advance to establish coordinates, and the coordinates were observed at the same place of each well plate each time to ensure the accuracy of the experiment.

#### 2.2.8. Determination of Phosphatidylserine (PS) Content

The experimental grouping was the same as that in Section 2.2.5. After reaching the culture time, the supernatant was sucked off, washed twice with PBS, added with 100 *μ*L binding buffer and 10 *μ*L Annexin V-FITC, kept away from light for 30 mins at room temperature, and immediately carried out quantitative detection by flow cytometry, while taking a tube without Annexin V-FITC as negative control.

#### 2.2.9. Detection of Mitochondrial Membrane Potential

The experimental grouping was the same as that in Section 2.2.5. After reaching the culture time, the supernatant was sucked off, and JC-1 working solution with a concentration of 20 *μ*M was added and incubated in an incubator at 37°C for 30 mins in the dark, washed twice with PBS, and observed by laser confocal microscope.

#### 2.2.10. Observation of Intracellular ROS Level

The experimental grouping was the same as that in Section 2.2.5. After reaching the culture time, the supernatant was sucked off, washed once with PBS, added with DCFH-DA dye solution, observed under a fluorescence microscope, and performed fluorescence semiquantitative analysis on ROS with Image J software.

#### 2.2.11. Detection of Intracellular Nitric Oxide (NO) Level

The experimental grouping was the same as that in Section 2.2.5. After the repair time was reached, the supernatant in the 6-well plate was sucked off, 1 mL DAF-FM DA diluent was added into each well for incubation in the dark at 37°C incubator for 30 mins, and the cells were washed three times with PBS to fully remove DAF-FM DA that did not enter the cells. The 6-well plate was observed under an inverted fluorescence microscope, and finally, fluorescence semiquantitative analysis of NO was performed using ImageJ software.

#### 2.2.12. Detection of Autophagy

The experimental grouping was the same as that in Section 2.2.5. After reaching the action time, the cells were digested and collected by pancreatin and then washed twice by adding a proper amount of 1× wash buffer. Then, 1 mL of 10% monodansylcadaverine (MDC) dye was added to each well and incubated in an incubator for 30 mins in the dark. After washing twice by wash buffer, the autophagy level of cells was quantitatively analyzed by flow cytometry.

#### 2.2.13. Calcein AM/PI Staining of Living/Dead Cells

The experimental grouping was the same as that in Section 2.2.5. After reaching the culture time, the supernatant was sucked off, and the cells were washed once with PBS. Then, 1 mL of calcein AM/PI detection working solution was added, and incubated for 30 mins at 37°C in the dark. After the incubation, Hoechst 33342 was used for staining for 10 mins, and the cells were detected and observed by laser confocal microscope (calcein AM is green fluorescence; PI is red fluorescence; Hoechst 33342 is blue fluorescence).

#### 2.2.14. Detection of Apoptosis and Necrosis

The experimental grouping was the same as that in Section 2.2.5. After reaching the culture time, the cells were digested with 0.25% trypsin, centrifuged (1000 rpm, 5 min) to collect the cells, which were mixed with 100 *μ*L binding buffer, followed by 2.5 *μ*L Annexin V-FITC and 5 *μ*L PI staining solution, and incubated for 15 mins in the dark at room temperature. Then, 200 *μ*L binding buffer was added, mixed evenly, transferred to a flow tube, and detected by flow cytometry.

#### 2.2.15. Detection of Cell Cycle

The experimental grouping was the same as that in Section 2.2.5. After reaching the culture time, the cells were collected and resuspended in 300 *μ*L PBS, slowly added with 700 *μ*L precooled anhydrous ethanol while shaking, and fixed at 4°C overnight in the dark. After centrifugal washing, the cells were resuspended in 500 *μ*L PBS. After centrifugation, the samples were stained with 200 *μ*L PI dye solution for 15 mins in an incubator at 37°C, filtered, and detected on the computer.

#### 2.2.16. Statistical Analysis

The data obtained in this experiment is expressed as average ± standard deviation (*x̅*±SD). SPSS 13.0 software (SPSS Inc., Chicago, IL, USA) was used to statistically analyze the experimental results, and the Tukey test was used to analyze the differences between the experimental groups and the control group. *P* < 0.05 means there are significant differences. *P* < 0.01 represents a very significant difference. *P* > 0.05 means no significant difference.

## 3. Results

### 3.1. Preparation and Characterization of CDSPs

#### 3.1.1. Preparation of CDSPs and -COOH Content Detection

DSP0 was subjected to bimolecular nucleophilic substitution reaction with different concentrations of chloroacetic acid under alkaline conditions, and the derivative polysaccharides with different degrees of carboxymethylation (CDSPs) were obtained. The content of -COOH in these polysaccharides was determined using conductometric titration (Table 1). Results showed that with increasing chloroacetic acid concentration, the contents of -COOH in CDSPs increased from 1.17% to 7.45% (CDSP1), 12.2% (CDSP2), and 17.7% (CDSP3).

The molecular weight of DSP0 measured through the viscosity method was 9.68 kDa, whereas those of CDSPs decreased slightly (8.76–9.40 kDa) because the carboxymethylation reaction was carried out in heated and strongly alkaline solution, at which time the polysaccharide was degraded to a certain extent [46]. Yang and Montgomery and Chiku et al. [47, 48] thought that the alkaline degradation of polysaccharide is a process of gradual “peeling” of the reducing end group, and after the alkali attacks the reducing end group of polysaccharide, the Lobry de Bruyn–van Ekenstein transformation occurs. The residue is converted into hydroxy acid. The glycosidic bond is broken due to the degradation of the reducing terminal.

#### 3.1.2. DSP Monosaccharide Composition Determination

The monosaccharide content of DSP was quantified using ion chromatography (Figures 1(k) and 1(l)), and results showed that DSP contained 98.24% glucose (Table 2), 0.64% galacturonic acid, 0.50% arabinose, 0.33% galactose, 0.18% rhamnose, 0.05% xylose, and 0.05% mannose. DSP molecules were blocks of glucose-linked residues.

#### 3.1.3. FT-IR Characterization

The FT-IR spectra of the four CDSPs with different -COOH contents are shown in Figure 1(a), and the major absorption peaks are presented in Table 3. The infrared characteristic absorption peaks of the four polysaccharides were similar, indicating that the carboxymethylation reaction had no significant effect on the overall structure of the polysaccharide. The absorption peaks at about 3397.9 and 2929.5 cm^−1^ were attributed to the telescopic vibrations of O-H and C-H bonds, respectively. The absorption peaks at about 1604.3 and 1417.4 cm^−1^ corresponded to the C=O asymmetric and C-O telescopic vibrations of -COOH. The absorption peak at about 1024.2 cm^−1^ indicated that the monosaccharide contained a pyranose ring [49].

Compared with those of DSP0, the absorption peaks of the three CDSPs at about 1326 cm^−1^ were significantly enhanced, and the peaks were attributed to the methylene stretching vibration [49], suggesting that the carboxyl and methylene (-CH_2_COOH) groups were successfully introduced after the carboxymethylation reaction. In addition, the intensity of this absorption peak was positively correlated with the content of -COOH in the polysaccharide, and the intensity of this peak was also the strongest on CDSP3 with the highest content of -COOH (17.7%, Figure 1(b)). This finding proved a successful carboxymethylation reaction.

#### 3.1.4. ^1^H NMR and ^13^C NMR Characterization

In ^1^H NMR, the chemical shift at *δ*_*H*_ 4.70 ppm was attributed to the D_2_O solvent. According to Guo et al. [50], the chemical shifts of the NMR spectra of monosaccharides, oligosaccharides, and polysaccharides are all within *δ*_*H*_ 1.0–6.0 ppm, whereas *δ*_*H*_ 4.3–6.0 ppm is considered to be related to anomeric protons, and *δ*_*H*_ 3.2–4.2 ppm is considered to be related to protons H-2 to H-6 on C-2 to C-6. According to Wang et al. [51], the regions of *δ*_*H*_ 5.0–5.8 and 4.4–5.0 ppm indicate the existence of *α* and *β* anomeric proton configurations, respectively, in polysaccharides. The ^1^H signals at *δ*_*H*_ 5.33, 5.32, and 4.89 (Figure 1(c)), 5.56, 5.33, and 4.89 (Figure 1(d)), 5.59, 5.36, 5.35, and 4.88 (Figure 1(e)) and 5.69, 5.46, and 4.89 ppm (Figure 1(f)) were designated as the anomeric proton (H-1) in the polysaccharides DSP0, CDSP1, CDSP2, and CDSP3, respectively. DSP0, CDSP1, CDSP2, and CDSP3 all had the *α*-configuration and *β*-configuration, but their *α*-configuration and *β*-configuration strengths gradually weakened. According to Liu et al. [52], the ^1^H signal of *δ*_*H*_ 0.8–1.4 ppm in the ^1^H NMR spectrum was usually designated as the proton signal of -CH_3_ (C-6) for deoxysugar. Thus, the ^1^H signal at *δ*_*H*_ 1.10 ppm (Figures 1(c)–1(f)) was designated as the proton of C6-CH_3_ (H-6) in the polysaccharide. In accordance with the component analysis of monosaccharide in polysaccharide and given that unlike glucose, rhamnose had a -CH_3_ group, the ^1^H signal of *δ*_*H*_ 1.10 ppm was the characteristic absorption peak of rhamnose.

In the ^13^C NMR spectrum, the component analysis of monosaccharide in polysaccharide showed that DSP was largely composed of glucose. Thus, Table 4 is assigned to the signal of main chain [21]. After carboxymethylation modification, the above signals of the DSP main chain remained, indicating that the main structure of DSP in its chemically modified derivatives was preserved. Given that the anomeric carbon atom signal was resonant in the range of *δ*_C_ 95.0–110 ppm [51], the resonance in the range of *δ*_C_ 97.0–101 ppm was attributed to the anomeric carbon in the *α*-configuration, and the resonance in the range of *δ*_C_ 103–107 ppm was attributed to the anomeric carbon in the *β*-configuration. Thus, the resonances at *δ*_C_ 103.66, 99.75, 99.60, and 95.89 (Figure 1(g)), 102.75, 99.48, and 96.99 (Figure 1(h)), 103.48, 99.30, and 96.91 (Figure 1(i)), and 102.49, 97.84, and 96.33 ppm (Figure 1(j)) were all attributed to the anomeric carbon signal, where DSP0, CDSP1, CDSP2, and CDSP3 all had the *α*-configuration and *β*-configuration. These findings were consistent with ^1^H NMR results. The electron density of the carbon at the substituted position increases with the substitution of carboxymethyl, resulting in the chemical shift of the substituted carbon to the low-field shift. These chemical shift changes indicate that the carboxymethyl group on the directly bonded carbon atom is replaced, and the electron density of these proton atoms is slightly increased [53]. Since the ether oxygen atom in the group has two unpaired electrons, which directly shield the connected carbon atom, the carboxymethyl substitution pair-substituted cyclic carbon only causes a small ^13^C chemical shift to a low field [54]. Liu et al. [10] indicated that the absence of *δ*_C_ 83.0–88.0 ppm signal in the ^13^C NMR spectrum reflected that all sugar residues of DSP and CDSPs existed in the pyranose form. The resonance around *δ*_C_ 70.0–85.0 ppm was designated as the signal of -CHOH (C-2, C-3, and C-4) on the sugar oxygen ring, where the peak signal was concentrated. According to Velichko et al. [55], the resonance around *δ*_C_ 62.0 ppm was -CH_2_OH (C-5 or C-6), and the resonance less than *δ*_C_ 20.0 ppm was designated as -CH_3_ (C-6) on methyl pentose. Therefore, the resonances at *δ*_C_ 60.74 and 60.45 (Figure 1(g)), 60.37 and 57.41 (Figure 1(h)), 60.32 and 57.42 (Figure 1(i)), and 60.22 and 57.43 ppm (Figure 1(j)) were attributed to -CH_2_OH (C-5 or C-6). Resonances at *δ*_C_ 17.96 (Figure 1(g)), 16.79 (Figure 1(h)), 16.80 (Figure 1(i)), and 16.82 ppm (Figure 1(j)) were attributed to -CH_3_ (C-6) on the methyl pentose. The absorption signal of carboxyl (-C=O) characteristic peak in carboxymethyl (-CH_2_COO^−^) could be observed in the range of *δ*_C_ 176–180 ppm [56], whereas the absorption signal of methylene carbon (-CH_2_-) characteristic peak in carboxymethyl (-CH_2_COO^−^) could be observed in the range of *δ*_C_ 70–71 ppm. Therefore, the resonance at *δ*_C_ 71.51 and 177.69 (Figure 1(h)), 71.60 and 177.69 (Figure 1(i)), and 70.40 and 177.64 ppm (Figure 1(j)) confirmed the carboxymethylated structure.

### 3.2. Antioxidant Activity of CDSPs

#### 3.2.1. Hydroxyl Radical (•OH) Scavenging Ability

**Figure 2:**
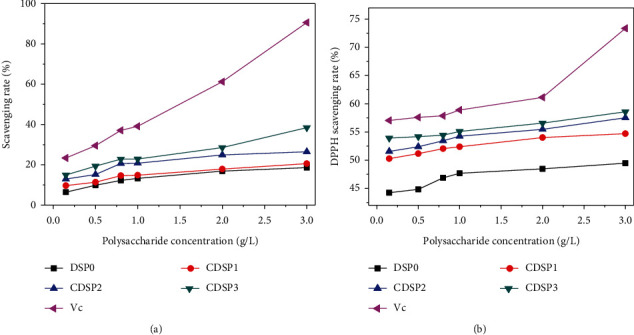
Comparison of antioxidant activities of CDSPs with different –COO^−^ contents. (a) The ability to scavenge •OH radicals; (b) DPPH scavenging activity.

•OH has extremely strong oxidation ability, which can lead to cell damage [57]. The in vitro •OH scavenging capacity of CDSPs is shown in Figure 2(a). With increasing CDSP concentration, the ability to remove •OH increased. At the same concentration, a high -COOH content resulted in increased ability of scavenging •OH.

#### 3.2.2. DPPH Radical Scavenging Ability

DPPH is a stable free radical and has been widely used to evaluate the antioxidant activity of natural compounds [58]. Results are shown in Figure 2(b). With increased CDSP concentration, the DPPH free radical scavenging rate increased. At the same concentration, a high –COOH content resulted in strong ability of scavenging DPPH, and the highest scavenging rate of CDSP3 reached 74.73%.

### 3.3. Cytotoxicity of CDSPs and Its Repairing Effect on Damaged Cell Vitality

**Figure 3:**
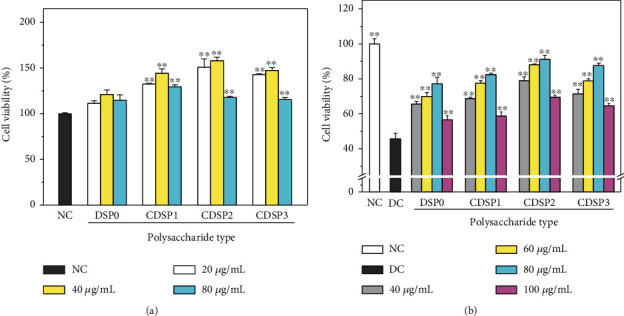
Cytotoxicity of CDSPs with different –COO^−^ contents (a) and its efficacy in repairing the viability of damaged HK-2 cells (b). NC: normal control group; DC: damage control group; oxalate concentration: 2.8 mM; injury time: 3 h; repair time: 12 h. Compared with the DC, ^∗^*P* < 0.05; ^∗∗^*P* < 0.01.

The four CDSPs had no toxicity to HK-2 cells (Figure 3(a)) and promoted cell proliferation at varying degrees.

CDSPs showed significant repair effects on HK-2 cells damaged by oxalate (Figure 3(b)). After being repaired by DSPs at the concentration of 40–100 *μ*g·mL^−1^ for 12 h, the cell viability increased from 45.63% in the DC group to 56.54%–91.18%. The lowest repair degree was 56.54% ± 2.80% in the DSP0 group with 100 *μ*g·mL^−1^, and the highest repair degree was 91.18% ± 1.33%, which was observed in the CDSP2 group 80 *μ*g·mL^−1^.

At the same concentration, the repairing abilities of polysaccharides followed the order DSP0 < CDSP1 < CDSP3 < CDSP2. The CDSP2 with medium -COOH content had the strongest repair ability. For the same polysaccharide, the repair ability was the highest at the concentration of 80 *μ*g·mL^−1^, and the repair ability decreased at concentration greater or less than 80 *μ*g·mL^−1^. When the polysaccharide content is low, the maximum repair effect has not been achieved. But when the concentration of CDSPs is too high, massive CDSP molecules will cover on cell surface, and it will affect cellular normal respiration and increase extracellular osmotic pressure, and finally lead cell activity decreased [59, 60].

### 3.4. Hematoxylin–Eosin (HE) Staining to Observe the Repair of Cell Morphology by CDSPs

HK-2 is composed of negatively charged acidic substances in the nucleus and has strong affinity with hematoxylin trioxide, an oxide of the alkaline dye hematoxylin with positive charge. Cytoplasm has a strong affinity for eosin, an acid dye with negative charge, because it contains alkaline substances with positive charge. Therefore, after HE staining, the nucleus was dyed blue–purple, and the cytoplasm was dyed red, pink, or orange–red.

**Figure 4:**
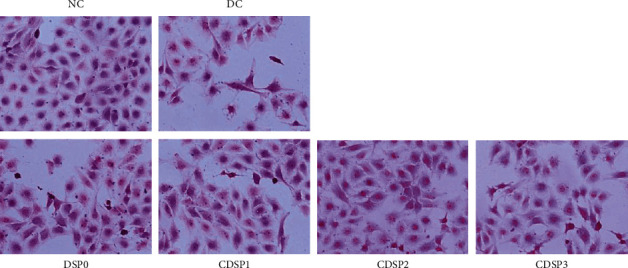
Cell morphology of damaged HK-2 cells repaired by CDSPs with different –COO^−^ contents. Polysaccharides concentration: 80 *μ*g·mL^−1^; oxalate concentration: 2.8 mM; injury time: 3 h; repair time: 12 h; scale bars: 50 *μ*m.

As shown in Figure 4, normal cells without oxalate damage were plump in shape and had many cells. In the DC group, the cell morphology shrank and was disordered. The cell membrane was ruptured, and the number of cells was significantly minimal.

After repair by CDSPs, the cell morphology was gradually intact, and the number was gradually increased with decreasing number of cells with cell membrane rupture. The cell state after repair by CDSP2 was close to that of normal cells.

### 3.5. Repair of the HK-2 Cytoskeleton by CDSPs

**Figure 5:**
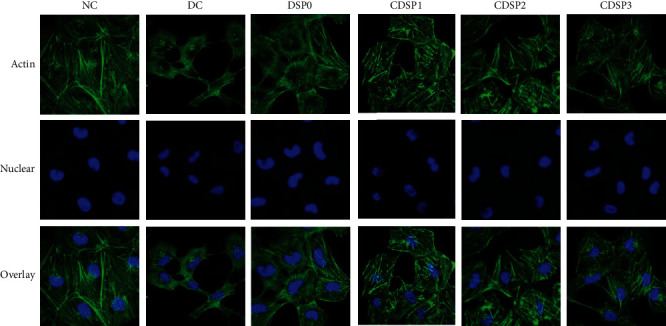
Cytoskeletal changes of CDSPs with different –COO^−^ contents after repairing damaged HK-2 cells. Polysaccharide concentration: 80 *μ*g·mL^−1^; oxalate concentration: 2.8 mM; injury time: 3 h; repair time: 12 h.

The changes in the HK-2 cytoskeleton before and after CDSP repair with different -COOH contents were observed using laser scanning confocal microscopy (Figure 5). The skeleton of normal cells was intact, and evident actin microfilaments were observed, and the nucleus was plump and spherical. However, after being damaged by oxalate, the cytoskeleton was severely deformed, the distribution of actin was disordered, and the nucleus shrank. After the damaged cells were repaired by CDSPs, the cell morphology was gradually plump, the actin was gradually clear, and the morphology of the nucleus was also improved, among which the cytoskeleton repaired by CDSP2 was the most complete.

### 3.6. Promoted Cell Healing by CDSPs

**Figure 6:**
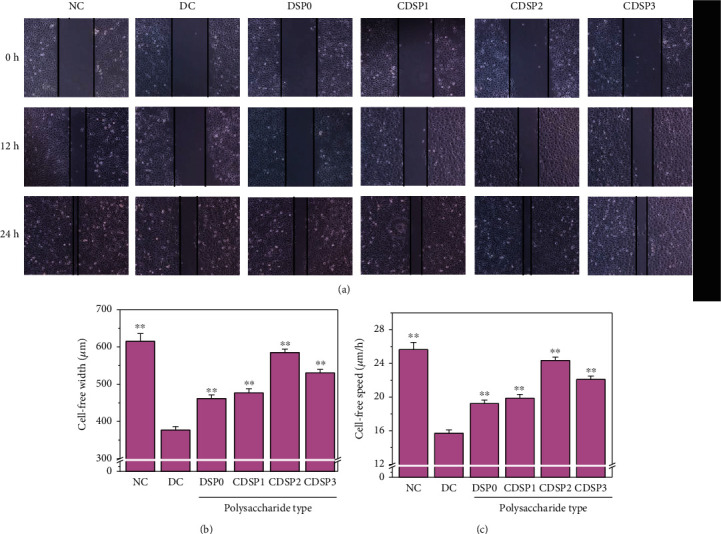
Healing ability of CDSPs with different -COO^−^ content after repairing damaged HK-2 cells. (a) Cell healing observed under a common microscope; (b) histogram of cell healing width within 24 h; (c) histogram of cell healing rate within 24 h. Oxalate concentration: 2.8 mmol/L, injury time: 3 h; polysaccharide concentration: 80 *μ*g·mL^−1^; injury time: 3 h; repair time: 12 h; scale bars: 200 *μ*m. Compared with the DC, ^∗^*P* < 0.05; ^∗∗^*P* < 0.01.

Microscopic observation (Figure 6(a)) showed that the cells in the normal group migrated quickly after scratches, and the cells in scratches grew quickly to close to healing after 24 h with a healing rate of 25.64 ± 0.80 *μ*m · h^−1^. The healing rate of DC group scratches was the slowest (15.70 ± 0.48 *μ*m · h^−1^).

After the damaged cells were repaired by each polysaccharide for 24 h, the cell healing width (Figure 6(b)) and healing rate (Figure 6(c)) of the scratch area were increased to different degrees, and amplitudes followed the order NC > CDSP2 > CDSP3 > CDSP1 > DSP0 > DC. The healing rate of CDSP2 was closest to that of the control.

### 3.7. Reduce Eversion of Phosphatidylserine (PS) by CDSPs

**Figure 7:**
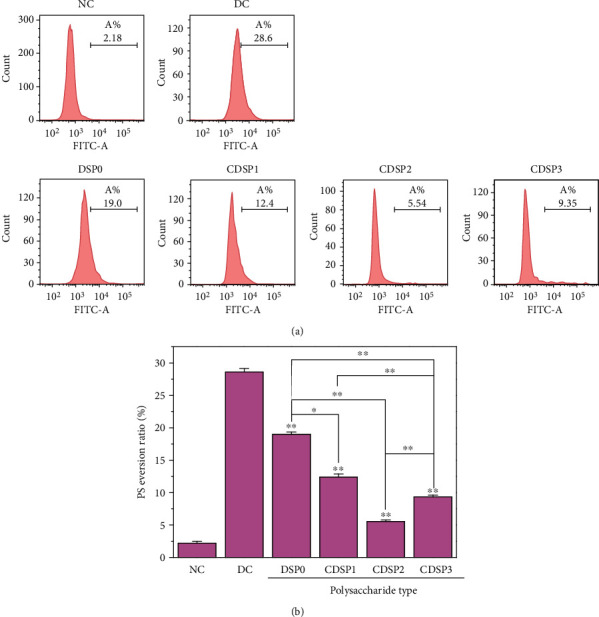
Detection of PS eversion after repair of damaged HK-2 cells by CDSPs with different –COO^−^ contents. (a) Histogram of PS eversion detected by flow cytometry; (b) quantitative histogram of PS eversion. Polysaccharide concentration: 80 *μ*g·mL^−1^; oxalate concentration: 2.8 mM; injury time: 3 h; repair time: 12 h. Compared with the DC, ^∗^*P* < 0.05; ^∗∗^*P* < 0.01.

The PS of normal cells is usually located in the inner leaf of the plasma membrane. However, in apoptotic cells, PS transfers from the inner leaf to the outer leaf of plasma membrane, thus being exposed to the external environment of cells. As shown in Figure 7, the PS eversion of damaged cells (28.6%) was much larger than that of normal cells (2.18%). However, after the damaged cells were repaired by CDSPs, their PS eversion decreased (19.0%–5.54%, Figure 7(b)). The PS eversion after CDSP2 repair (5.54%) was close to that in the normal group, and the repair effect was the best.

### 3.8. Enhanced Mitochondrial Membrane Potential by CDSPs

**Figure 8:**
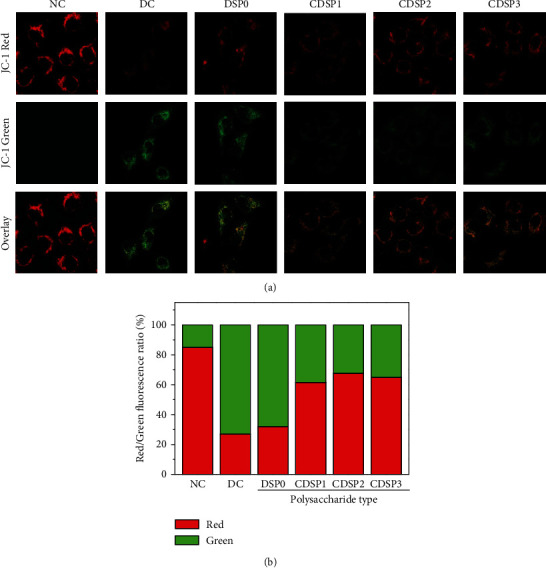
Mitochondrial membrane potential detection of DSPs with different carboxyl contents after repairing damaged HK-2 cells. (a) Laser scanning confocal microscope was used to observe the changes of mitochondrial membrane potential. (b) Histogram of red–green fluorescence intensity. Concentration of polysaccharide: 80 *μ*g·mL^−1^. Oxalate concentration: 2.8 mM; injury time: 3 h; repair time: 12 h.

The cationic lipophilic dye JC-1 can be used as a fluorescent probe for detecting mitochondrial membrane potential, and its red–green fluorescence transition represents the change in membrane potential. As shown in Figure 8, almost no green fluorescence was observed in normal cells. Thus, the mitochondrial membrane potential was high, and JC-1 was gathered in the matrix of mitochondria, forming polymer and emitting red fluorescence. The green fluorescence of damaged cells was relatively strong, showing only weak red fluorescence, indicating that its membrane potential was low and that JC-1 was mostly monomer. After the damaged cells were repaired by CDSPs, the red fluorescence of the cells showed different degrees of enhancement (Figure 8(b)), and the CDSP2 group had the best effect.

### 3.9. Reducing Intracellular ROS Levels by CDSPs

**Figure 9:**
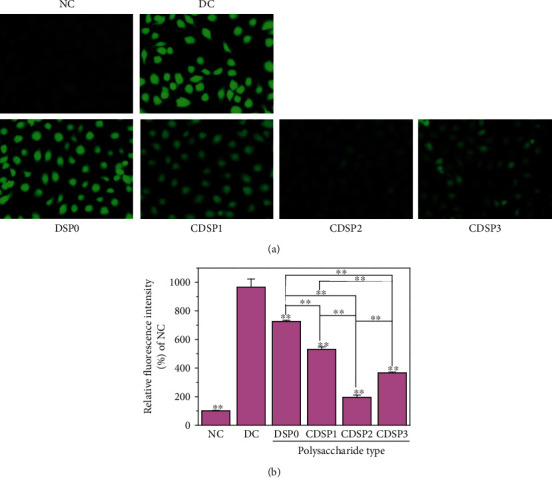
ROS level detection. (a) ROS fluorescence photograph; (b) histogram of relative fluorescence intensity: polysaccharide concentration, 80 *μ*g·mL^−1^; oxalate concentration, 2.8 mM; injury time: 3 h; repair time: 12 h; scale bars: 50 *μ*m. Compared with the DC, ^∗^*P* < 0.05; ^∗∗^*P* < 0.01.

The proliferation of intracellular ROS or the decline in endogenous antioxidant capacity may lead to the imbalance of cell oxidation and antioxidant function, resulting in oxidative stress [61]. As shown in Figure 9, normal cells basically had no green fluorescence, indicating that the ROS level in cells was low. However, the green fluorescence of HK-2 cells was significantly enhanced by oxalate after oxidative damage, which indicated that oxalate caused severe oxidative damage.

After the damaged cells were repaired by CDSPs for 12 h, their green fluorescence appeared to be weakened to different extents, and CDSP2 with a -COOH content of 12.2% had the best scavenging effect on ROS in cells (Figure 9(b)).

### 3.10. Reduction of Intracellular Nitric Oxide (NO) Level by CDSPs

Intracellular NO can induce diseases, such as cancer, neurodegenerative diseases, and stroke. The peroxynitrite anion (ONOO^–^) is the product of the reaction of NO with a superoxide, also known as RNS. ROS and RNS play a key role in various signaling and pathological processes, but the excessive production of ROS and RNS caused by exogenous stimulation is harmful, which can induce the oxidation of DNA, protein, or lipid and lead to cell death [62].

DAF-FM DA easily diffused into cells and released DAF-FM in the cytoplasm by the action of cell lactonase. DAF-FM without fluorescence was oxidized by NO to form DAF-FM triazole, and its green fluorescence intensity was increased by about 160 times.

**Figure 10:**
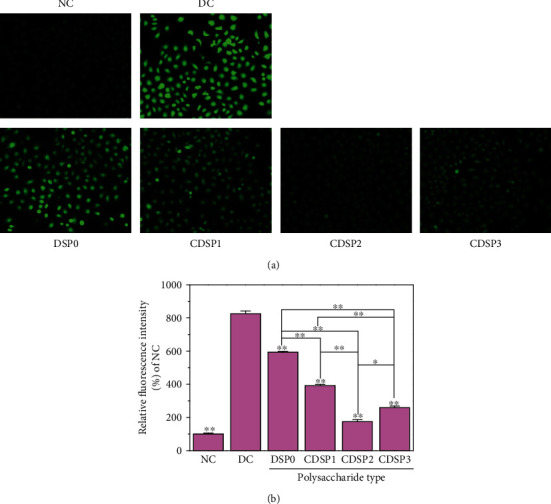
Detection of nitric oxide (NO) level. (a) NO fluorescence photograph; (b) histogram of relative fluorescence intensity. Concentration of polysaccharide: 80 *μ*g·mL^−1^; oxalate concentration: 2.8 mM; repair time: 12 h; scale bars: 200 *μ*m. Compared with the DC, ^∗^*P* < 0.05; ^∗∗^*P* < 0.01.

Under a fluorescence microscope, the green fluorescence of normal cells was weak (Figure 10), suggesting minimal NO in the cells. However, the green fluorescence of cells damaged by oxalate was significantly enhanced. Oxalate caused severe oxidative damage to cells, leading to increased intracellular NO, improved level of oxidative stress in cells, and further exacerbated cell damage. After the damaged cells were repaired by CDSPs, the green fluorescence of the cells gradually weakened, the level of NO decreased, and the CDSP2 group was the closest to the normal group.

### 3.11. Reduced Autophagy Level of Cells by CDSPs

Autophagy, a form of programmed cell death, is regarded as an adaptive response to survival. Autophagy, which plays a key role in maintaining the development and steady state of cells, can remove damaged organelles and protein or reduce the accumulation of toxic products [63]. However, unrestricted autophagy can also promote cell death and morbidity [64]. Under normal physiological conditions, autophagy is maintained at a low level. When cells are treated with harmful substances, the autophagy level significantly increases [65].

**Figure 11:**
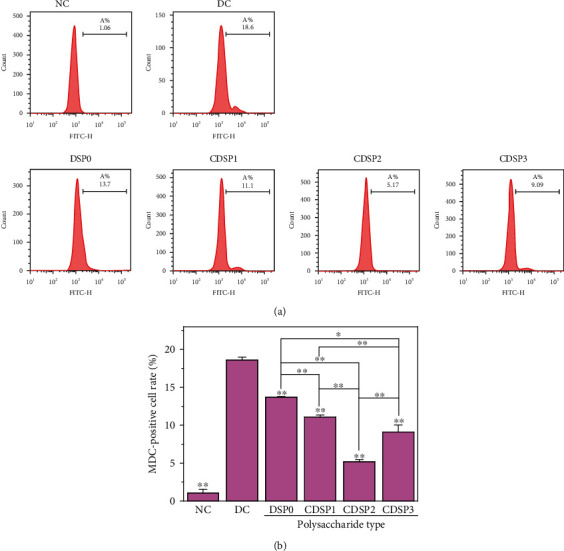
Detection of autophagy after repairing damaged HK-2 cells with CDSPs with different –COO^−^ content. (a) Detection of autophagy histogram by flow cytometry; (b) quantitative histogram of autophagy. Polysaccharide concentration: 80 *μ*g·mL^−1^; oxalate concentration: 2.8 mM; injury time: 3 h; repair time: 12 h. Compared with the DC, ^∗^*P* < 0.05; ^∗∗^*P* < 0.01.

MDC is an eosinophilic fluorescent dye and is used as a specific labeling stain for detecting autophagy. The autophagy results detected by flow cytometry are shown in Figure 11. The autophagy of normal cells was low (1.06%), whereas that of damaged cells increased significantly (18.6%).

After the damaged cells were repaired by CDSPs, their autophagy levels decreased (Figure 11(b)). Along with increased -COOH content in polysaccharides, the proportion of MDC-positive cells was decreased first and then increased. The lowest level in the CDSP2 group (5.17%) indicated that the autophagy level of cells after CDSP2 repair was the lowest, which was conducive to maintaining the homeostasis of the cellular environment and reducing the risk of kidney calculi formation.

### 3.12. Living/Dead Cell Staining

Calcein AM is a substance that does not fluoresce. Calcein AM can enter living cells containing esterase to produce calcein with strong green fluorescence, and dead cells produce little calcein. PI can only dye dead cells, whose cell membrane integrity is destroyed and shows red fluorescence [66]. The Hoechst 33342 can penetrate the cell membrane into normal and apoptotic cells and combine with intracellular DNA to show blue fluorescence. After staining, the fluorescence of apoptotic cells is significantly enhanced compared with that of normal cells [67].

**Figure 12:**
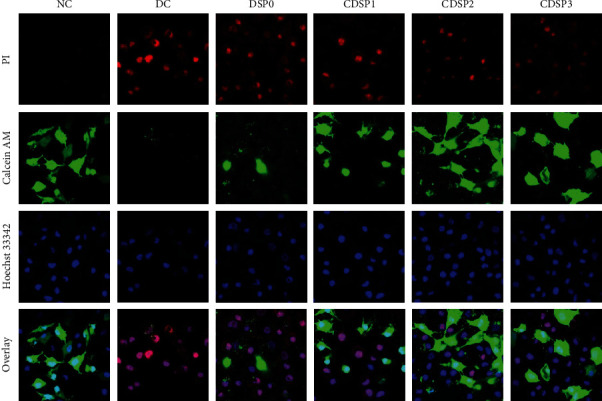
Staining of living/dead cells after repair with CDSPs containing different –COO^−^ contents (calcein/PI staining). Normal cells showed weak red fluorescence + weak blue fluorescence + strong green fluorescence, apoptotic cells showed weak red fluorescence + strong blue fluorescence + weak green fluorescence, and necrotic cells showed strong red fluorescence + strong blue fluorescence + weak green fluorescence. Oxalate concentration: 2.8 mM; polysaccharide concentration: 80 *μ*g·mL^−1^; injury time: 3 h; repair time: 12 h.

As shown in Figure 12, normal cells showed almost no red fluorescence, and most cells were stained green with strong fluorescence intensity, indicating that the proportion of living cells was extremely high. The number of cells stained red in the DC group was significantly increased, and the red fluorescence was significantly enhanced. The green fluorescence was weak, suggesting that cells died at varying degrees.

After damaged cells were repaired by CDSPs, the blue fluorescence intensity of the nuclei of normal cells was weaker than that of dead cells, and the number of dead cells was less than that of the DC group, indicating that the number of living cells was increased after polysaccharide repair. The green fluorescence intensity of CDSP2 group was strongest, the number of living cells was the largest, and the number of dead cells was lowest.

### 3.13. CDSP Treatment Reduced Cell Death

**Figure 13:**
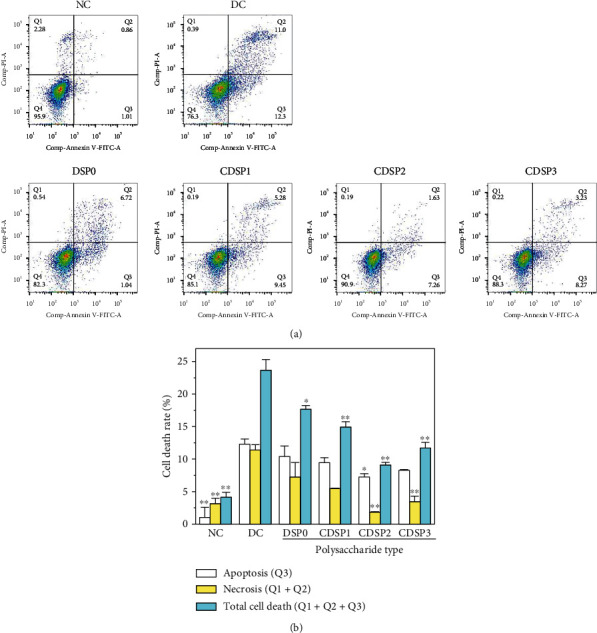
Detection of apoptosis and necrosis after repair of damaged HK-2 cells with CDSPs of different –COO^−^ contents. (a) Flow scatter plot of apoptosis and necrosis; (b) quantitative histogram of apoptotic necrosis. The white column was apoptotic cells, and the ordinate was *Q*3. The yellow column is necrotic cell, and the vertical coordinate is *Q*1 + *Q*2; the green column was taken as the total dead cells, and the ordinate was *Q*1 + *Q*2 + *Q*3. The concentration of polysaccharide was 80 *μ*g·mL^−1^. Oxalate concentration: 2.8 mM; injury time: 3 h; repair time: 12 h. Compared with the DC, ^∗^*P* < 0.05; ^∗∗^*P* < 0.01.

The ratio of apoptosis and necrosis was further detected by Annexin V–FITC/PI double-staining method (Figure 13). The ratio of apoptosis and necrosis of normal cells was low, with only 4.15% dead cells. In the DC group, the apoptosis rate (*Q*2 + *Q*3 = 23.3%) increased significantly, and the total proportion of dead cells (*Q*1 + *Q*2 + *Q*3) reached 23.69%.

After the damaged cells were repaired by CDSPs, the total proportion of dead cells decreased significantly (9.08%–17.66%, Figure 13(b)), and CDSP2 was closest to the normal group.

### 3.14. CDSP Treatment Restored Cell Cycle Progress

**Figure 14:**
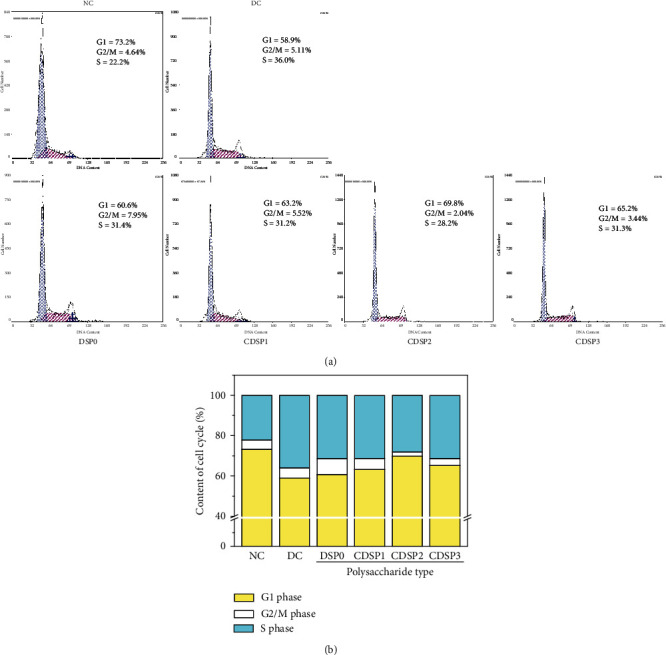
Cell cycle analysis of CDSPs with different –COO^−^ contents after repairing damaged HK-2 cells. (a) Cell cycle diagram; (b) quantitative histogram of cell cycle: polysaccharide concentration: 80 *μ*g·mL^−1^; oxalate concentration: 2.8 mM; injury time: 3 h; repair time: 12 h.

Cell cycle changes were detected by flow cytometry to detect the repair effect of CDSPs on damaged DNA (Figure 14). The proportion of cells in the S phase in the normal group was 22.2%, whereas that in the DC group increased to 36.0%, indicating that oxalate caused the retention of the cell cycle in the S phase and was accompanied by decreased number of cells in the G1 phase (from 73.2% to 58.9%). The oxalate injury inhibited the synthesis of cell DNA.

After the damaged cells were repaired by CDSPs, the proportion of cells in the G1 phase increased, and the increase in the CDSP2 repair group (from 58.9% to 69.8%) was the most significant. At the same time, the proportion of cells in the S phase decreased after repair with CDSPs, in which the proportion in the CDSP2 repair group decreased from 36.0% to 28.2% (Figure 14(b)). Polysaccharide repair normalized the cell cycle process and promoted cell proliferation, and the proportion of each cycle of cells after CDSP2 repair was closest to that of normal cells.

## 4. Discussion

### 4.1. Carboxymethylation Improves the Antioxidant Activity of DSP

Plant polysaccharides have extensive antioxidant activity and play a vital role in resisting cell oxidative damage [68]. Carboxymethylation refers to the introduction of carboxymethyl groups (-CH_2_COOH) into the polysaccharide chain. Many hydroxyl groups were distributed in the main and side chains of polysaccharides. Under the alkaline condition of NaOH, the 1° or 2° alcohol groups in the sugar unit reacted with OH^−^ to generate alkoxide groups, which improved the nucleophilicity. In alkaline environment, negative oxygen anion reacts with *α*-C of chloroacetic acid to produce carboxymethyl polysaccharide [69]. In this paper, we successfully carboxymethylated DSP0, and the -COOH content of CDSP derivatives depended on the amount of chloroacetic acid.

The scavenging ability of CDSPs on •OH and DPPH radicals increased with increased -COOH content of polysaccharide (Figure 2), and the scavenging ability of CDSPs on DPPH radicals was more evident than on •OH (Figure 2(b)). Nuerxiati et al. [70] reported that carboxymethylated derivatives of *Orchis chusua* D. Don (Salep) polysaccharide (SP-C) exhibited stronger DPPH radical scavenging activity than phosphorylated derivatives of SP and sulfate derivatives of SP. SP-C has more lone pairs of electrons than other derivatives, and the lone pair electrons of carboxymethylated polysaccharides can provide electrons to free radicals or attract unpaired electrons of free radicals, so it is easy to capture free radicals, directly quench or inhibit free radicals, stop the chain reaction of free radicals, and play an antioxidant role. Alavi Rafiee et al. [71] reported that the proton of -COOH group dissociates from polar medium and changed into carboxylate anion (-COO^−^) and that the electron donor effect of -COO^−^ can reduce the dissociation energy of hydroxyl group, which is beneficial to hydrogen atom transfer and free radical scavenging. Second, carboxymethylation modification can improve the polarity and solubility of polysaccharides [72], which is beneficial to improve the molecular mobility and utilization ratio to a remarkable extent and further enhance its ability to scavenge free radicals [73]. Finally, carboxymethylated polysaccharides can also inhibit the generation of free radicals by chelating transition metal ions (such as Fe^2+^ and Cu^2+^) [74, 75], instead of directly scavenging free radicals, thus exerting its antioxidant activity.

### 4.2. Repair of Damaged HK-2 Cells by CDSPs

CDSPs reduced the ROS level in HK-2 cells damaged by oxalate (Figure 9) but also restored the mitochondrial membrane potential (Figure 8), thus reducing oxidative stress. Evidence showed that excessive ROS is an important cause of kidney calculi [61, 76]. The mitochondria are the main source of ROS production. When ROS is produced uncontrollably or the ability of endogenous antioxidants is reduced, cell oxidative stress occurs [77], leading to mitochondrial dysfunction. Moreover, the release of proapoptotic factors from the mitochondria with lost membrane potential causes apoptosis and leads to kidney damage and the occurrence of calculi [61]. Zhu et al. [78] studied the estrogen receptor *β* and found that it can inhibit the formation of CaOx crystals by reducing oxalate biosynthesis in the liver and cell damage mediated by renal oxidative stress. Therefore, our results demonstrated the potential of CDSPs to inhibit oxidative stress and prevent gallstones.

In addition, CDSPs repaired the morphology and cytoskeleton of HK-2 cells (Figures 4 and 5), increased the cell viability (Figure 3) and the cell healing rate (Figure 6), and reduced the eversion of PS (Figure 7), thus reducing the cell damage induced by oxalate. Actin, a cytoskeleton protein, plays an important role in the establishment and maintenance of cell morphology [79]. The disordered distribution of actin may cause various cell damages, such as mitochondrial dysfunction and neuronal death [80]. Cell healing rate is an important indicator to assess the ability of damaged cells to reestablish a complete tissue layer and restore normal function in the process of wound healing [81]. The eversion of PS on cell membrane can also show the degree of cell injury.

Results showed that CDSPs had good repair effect on oxalate-induced cell injury.

### 4.3. Maintenance of the Physiological State of Cells and Reduction in Cell Death by CDSPs

Knowledge about the role of autophagy in the occurrence and development of CaOx kidney calculi is lacking. Whether autophagy protects or damages renal tubular epithelial cells depends on different pressure factors [82–85]. Flow cytometry and confocal microscopy results showed that CDSPs reduced the increase in autophagy level induced by oxalate (Figure 11) and finally reduced cell death caused by injury (Figures 12 and 13). Evidence showed that autophagy is associated with kidney disease [82]. Liu et al. [83] showed that CaOx crystals can induce autophagy by activating the ROS pathway, thus aggravating the damage of renal tubular epithelial cells. Crystal-induced HK-2 cell injury was alleviated after autophagy was inhibited by knocking down the autophagy gene with an autophagy inhibitor and a special siRNA. The overproduction of ROS is the driver of autophagy [82]. Xu et al. [85] proved that the TGF-*β*1 activates autophagy by producing ROS, which aggravates the apoptosis of renal tubular epithelial cells. Our results also showed that CDSPs reduced ROS level (Figure 9) and autophagy level (Figure 11).

**Figure 15:**
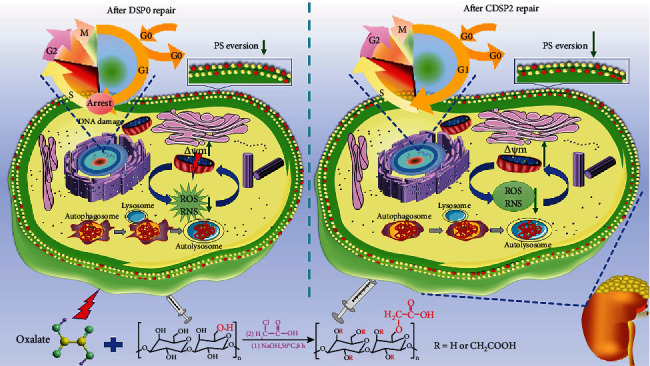
Mechanism of DSP0 and CDSP2 in repairing HK-2 cells damaged by oxalate.

On the basis of the results of this paper, we put forward the mechanism of CDSPs in repairing HK-2 cells damaged by oxalate (Figure 15). CDSPs reduce (1) oxidative stress and autophagy by improving cell vitality, reducing ROS production and restoring mitochondrial membrane potential and (2) apoptosis of HK-2 cells by repairing cell morphology and cytoskeleton damaged by oxalate, increasing cell healing rate, reducing PS eversion, and maintaining normal cell cycle.

### 4.4. Effect of -COOH Content in CDSPs on Biological Activity

#### 4.4.1. High Carboxyl Content in Polysaccharide Resulting in Strong Ability of Scavenging Free Radicals

Results in Figure 2 showed that a high -COOH content of CDSPs resulted in high capability of scavenging •OH and DPPH radicals. This finding might be related to the ionization of -COOH in aqueous solution, which increased the electron density of the sugar ring and increases the water solubility of the polysaccharide [86]. The proton of -COOH is easily dissociated in the polar medium and becomes the anion with electron donor effect, i.e., -COO^−^, which is conducive to the free radical removal based on the electron donor effect [71]. The increase in -COO^−^ content in the polysaccharide chain led to an increase in the electron density of the sugar ring. The chain conformation of -COO^−^ was more extended due to electrostatic repulsion, resulting in improved biological activity (including antioxidant activity) [18]. In addition, carboxymethylation-modified polysaccharides exhibit improved water solubility, facilitating their repair activity [14, 87, 88]. Wang et al. [89] also showed that after *Cyclocarya paliurus* polysaccharides are modified with different degrees of carboxymethylation, the polysaccharide with the highest degree of substitution (highest content of -COOH) has the highest antioxidant activity.

#### 4.4.2. CDSP2 with Medium -COOH Content Has the Strongest Ability to Repair Damaged HK-2 Cells

Although CDSP3 had the highest amount of antioxidant, the CDSP2 with medium -COOH content showed the best repair effect on damaged HK-2 cells. The reasons for the inconsistent rules between the two were as follows:

- The excessive -COOH content probably destroys the triple-helix structure of polysaccharides [75], and only the appropriate -COOH content is conducive to enhancing the chain conformation stability of polysaccharides. The spiral structure of polysaccharide has great influence on its biological activity. The chain with helical structure is easier to bind to the receptor on the cell surface, showing high biological activity [90]. For example, Cai and Zhang [91] found that when DS < 0.25, the chain conformation of CMCD was triple helix, while when DS > 0.25, the chain of CMCD was randomly coiled. This indicates that too high content of -COOH in polysaccharide will weaken the triple helix conformation of polysaccharide. Because the anion group (-COO^−^) is excessively increased, the repulsion between -COOH is enhanced, and the steric hindrance between polymer chains is enhanced, so that it will have a relatively expanded conformation, which makes it difficult for it to cross the cell membrane and enter the cell to exert its biological activity [92, 93]. Therefore, the repair effect of CDSP3 with the highest -COO^−^ content is not the best
- Although polysaccharides with good water solubility were beneficial to their antioxidant activities, the repair of damaged cells was not performed in aqueous solution. Therefore, polysaccharides with the best antioxidant activity were not necessarily the strongest in cell repair. In addition, although the relationship between the content of -COOH in CDSPs and its water solubility was not clear in this study, Theis et al. [14] showed that increased carboxymethylation (DS > 0.47) does not promote increased polysaccharide solubility, but the carboxymethylated (1–6)-*β*-d-glucan with the lowest degree of substitution (DS 0.32) shows high solubility (67.99 mg per 100 mL). Liu et al. [75] reported that carboxymethylated polysaccharides from *Catathelasma ventricosum* with moderate degree of substitution has the best antioxidant and antibacterial activities. Li et al. [18] also mentioned that moderate carboxymethylation is beneficial to improve the biological activity of polysaccharides, but excessive substitution by carboxyl or carboxymethyl is not beneficial. It was proposed that the introduction of -COOH groups may affect the polarity, conformation, or charge density of polysaccharides, which may lead to various changes in the scavenging activity. These results are consistent with the results obtained in this study
- Carboxymethylation modification resulted in the decrease of molecular weight of polysaccharides (Table 1). The water solubility of low-molecular weight polysaccharides is often increased, thus having high freedom [32, 33]. A small steric hindrance and large degree of freedom are beneficial to improve the antioxidant activity [27]
- The ability of polysaccharides to repair oxalate-damaged renal epithelial cells is dependent on the antioxidant activity of polysaccharides. The biological activity of polysaccharides is not a function of a single factor but is related to multiple structural characteristics of polysaccharides, such as active group content, molecular weight, and monosaccharide composition and configuration [10, 94]
- Many studies showed that as a drug, polysaccharides exert their protective or repair effects on cells by relying on different intracellular signaling pathways [95–97]. However, the relationship between the structure and activity of *D. styracifolium* polysaccharide and its role in the signaling pathway remain a mystery, and methods should be explored especially through in vivo experiments

## 5. Conclusion

Three kinds of derived polysaccharides, i.e., CDSP1, CDSP2, and CDSP3 with -COOH content of 7.45%, 12.2%, and 17.7%, were obtained through the carboxymethylation of DSP0. The carboxymethylation modification significantly enhanced the antioxidant activity and the ability to repair damaged cells of the polysaccharide in vitro. CDSPs can enhance the viability and self-healing ability of damaged HK-2 cells, restore cell morphology and skeleton, reduce ROS production and PS eversion, maintain mitochondrial integrity and normal cell cycle, and reduce autophagy level, thus effectively repairing oxalate-mediated cell injury. CDSP2 with medium -COOH content had the best effect in repairing damaged HK-2 cells. These results suggested that CDSPs may play a potential role in the treatment of CaOx kidney calculi.

## Figures and Tables

**Table 1:** Carboxymethylation conditions of *Desmodium styracifolium* polysaccharide and carboxyl content in the product.

Polysaccharide type	Molecular weight/kDa	Reaction t/h	Reaction T/°C	Isopropyl alcohol/mL	30%NaOH/mL	Chloroactic acid/g	Productive rate/%	Carboxyl content/%
DSP0	9.68	/	/	/	/	/	/	1.17 ± 0.10
CDSP1	8.76	8	50	12	15	1.041	34.58	7.45 ± 0.27
CDSP2	9.19	8	50	12	15	2.068	42.73	12.2 ± 0.17
CDSP3	9.40	8	50	12	15	3.012	39.47	17.7 ± 0.65

**Table 2:** Summary of information on standard curves for determination of monosaccharide composition content of *Desmodium styracifolium* polysaccharide.

Standard monosaccharides	Peak time	Slope	Degree of fitting	Percentage composition
Fuc	3.625	0.4282	0.99232	N
D-GalN	7.025	1.0393	0.99355	N
Rha	7.45	0.2586	0.99569	0.18%
Ara	7.759	0.4534	0.99235	0.50%
GluN	8.917	0.8971	0.99207	N
Gal	9.817	0.3453	0.99231	0.33%
Glc	11.267	0.6529	0.99171	98.24%
Xyl	13.234	0.6629	0.99537	0.05%
Man	13.867	0.3839	0.99873	0.05%
Fru	15.909	0.2134	0.99847	N
Rib	17.267	0.497	0.99825	N
Gal-UA	34.175	0.2381	0.99934	0.64%
Gul-UA	34.942	0.306	0.99827	N
Glc-UA	36.759	0.465	0.99869	N
Man-UA	39.025	0.154	0.99883	N

N: not detected.

**Table 3:** Characteristic FT-IR absorption peaks of *Desmodium styracifolium* polysaccharide before and after carboxylation.

Sample	–COOH content	Characteristic absorption peaks of groups/cm^−1^					
		-OH	-CH_2_	Asymmetric C=O	C-O	-CH_2_ due to the introduction of carboxymethylation	Sugar ring
DSP0	1.17%	3397.9	2929.5	1642.3	1416.8	—	1024.2
CDSP1	7.45%	3421.6	2928.3	1603.2	1420.7	1327.3	1019.1
CDSP2	12.2%	3420.1	2927.6	1604.3	1418.9	1325.9	1025.0
CDSP3	17.7%	3376.8	2927.9	1604.4	1422.4	1326.3	1049.9

**Table 4:** ^13^C NMR signal of *Desmodium styracifolium* polysaccharide backbone.

Monosaccharide/*δ*_C_	C-1	C-2	C-3	C-4	C-5	C-6
*α*-Glucose	103.66	75.74	76.39	69.63	76.17	62.5
*β-*Glucose	99.60	72.87	73.97	69.59	72.70	61.32

## Data Availability

All the data supporting the results were shown in the paper and can be applicable from the corresponding author.

## References

[1] Kittanamongkolchai W., Vaughan L. E., Enders F. T. (2018). The changing incidence and presentation of urinary stones over 3 decades. *Mayo Clinic Proceedings*.

[2] Sun Y., Kang J., Guan X., Xu H., Wang X., Deng Y. (2021). Regulation of endoplasmic reticulum stress on the damage and apoptosis of renal tubular epithelial cells induced by calcium oxalate crystals. *Urolithiasis*.

[3] Sun X. Y., Zhang H., Chen J. Y., Zeng G. H., Ouyang J. M. (2021). *Porphyra yezoensis* polysaccharide and potassium citrate synergistically inhibit calcium oxalate crystallization induced by renal epithelial cells and cytotoxicity of the formed crystals. *Materials Science and Engineering: C*.

[4] Ding T., Zhao T., Li Y. (2021). Vitexin exerts protective effects against calcium oxalate crystal-induced kidney pyroptosis *in vivo* and *in vitro*. *Phytomedicine*.

[5] Han J., Guo D., Sun X. Y., Wang J. M., Ouyang J. M., Gui B. S. (2019). Repair effects of astragalus polysaccharides with different molecular weights on oxidatively damaged HK-2 cells. *Scientific Reports*.

[6] Sun X. Y., Zhang H., Liu J., Ouyang J. M. (2019). Repair activity and crystal adhesion inhibition of polysaccharides with different molecular weights from red algae *Porphyra yezoensis* against oxalate-induced oxidative damage in renal epithelial cells. *Food & Function*.

[7] Liang L., Li L., Tian J. (2014). Androgen receptor enhances kidney stone-CaOx crystal formation via modulation of oxalate biosynthesis & oxidative stress. *Molecular Endocrinology*.

[8] Marneros A. G. (2021). Magnesium and calcium homeostasis depend on KCTD1 function in the distal nephron. *Cell Reports*.

[9] Peng Q. L., Li C. Y., Zhao Y. W., Sun X. Y., Liu H., Ouyang J. M. (2021). Protective effect of degraded Porphyra yezoensis polysaccharides on the oxidative damage of renal epithelial cells and on the adhesion and endocytosis of nanocalcium oxalate crystals. *Oxidative Medicine and Cellular Longevity*.

[10] Liu Y., Duan X., Zhang M. (2021). Extraction, structure characterization, carboxymethylation and antioxidant activity of acidic polysaccharides from *Craterellus cornucopioides*. *Industrial Crops and Products*.

[11] Wang Q., Niu L. L., Liu H. P., Wu Y. R., Li M. Y., Jia Q. (2021). Structural characterization of a novel polysaccharide from *Pleurotus citrinopileatus* and its antitumor activity on H22 tumor-bearing mice. *International Journal of Biological Macromolecules*.

[12] Li Y., Ran C., Wei K. (2021). The effect of *Astragalus* polysaccharide on growth, gut and liver health, and anti-viral immunity of zebrafish. *Aquaculture*.

[13] Chen S., Liu C., Huang X. (2020). Comparison of immunomodulatory effects of three polysaccharide fractions from *Lentinula edodes* water extracts. *Journal of Functional Foods*.

[14] Theis T. V., Santos V. A. Q., Appelt P. (2019). Fungal exocellular (1-6)-*β*-d-glucan: carboxymethylation, characterization, and antioxidant activity. *International Journal of Molecular Sciences*.

[15] Li C. Y., Liu L., Zhao Y. W., Chen J. Y., Sun X. Y., Ouyang J. M. (2021). Inhibition of calcium oxalate formation and antioxidant activity of carboxymethylated *Poria cocos* polysaccharides. *Oxidative Medicine and Cellular Longevity*.

[16] Sun X., Zhao C., Pan W., Wang J., Wang W. (2015). Carboxylate groups play a major role in antitumor activity of *Ganoderma applanatum* polysaccharide. *Carbohydrate Polymers*.

[17] Li Y., Yuan Y., Lei L. (2017). Carboxymethylation of polysaccharide from *Morchella angusticepes* Peck enhances its cholesterol-lowering activity in rats. *Carbohydrate Polymers*.

[18] Li J., Shang W. T., Si X. (2017). Carboxymethylation of corn bran polysaccharide and its bioactive property. *International Journal of Food Science & Technology*.

[19] Deng Q., Wang X., Chen H., Zhao C., Gong X., Zhou X. (2020). Structural characterization, modification and hepatoprotective effects of polysaccharide from *Mori Fructus*. *International Journal of Biological Macromolecules*.

[20] Wu Q., Qin D., Cao H., Bai Y. (2020). Enzymatic hydrolysis of polysaccharide from *Auricularia auricula* and characterization of the degradation product. *International Journal of Biological Macromolecules*.

[21] Chen Y., Zhang H., Wang Y., Nie S., Li C., Xie M. (2014). Acetylation and carboxymethylation of the polysaccharide from *Ganoderma atrum* and their antioxidant and immunomodulating activities. *Food Chemistry*.

[22] Chen F., Huang G., Huang H. (2021). Preparation, analysis, antioxidant activities *in vivo* of phosphorylated polysaccharide from *Momordica charantia*. *Carbohydrate Polymers*.

[23] Wu J., Zhang Y., Ye L., Wang C. (2021). The anti-cancer effects and mechanisms of lactic acid bacteria exopolysaccharides *in vitro*: a review. *Carbohydrate Polymers*.

[24] Chen X., Zhang L., Cheung P. C. (2010). Immunopotentiation and anti-tumor activity of carboxymethylated-sulfated *β*-(1->3)-d-glucan from *Poria cocos*. *International Immunopharmacology*.

[25] Xie H., Li J., Gao H. (2018). Total flavone of *Desmodium styracifolium* relieved apoptosis and autophagy of COM-induced HK-2 cells by regulating KIM-1 via p38/MAPK pathway. *Molecular and Cellular Biochemistry*.

[26] Duan S., Zhao M., Wu B. (2020). Preparation, characteristics, and antioxidant activities of carboxymethylated polysaccharides from blackcurrant fruits. *International Journal of Biological Macromolecules*.

[27] Hu S., Wang D., Zhang J. (2016). Mitochondria related pathway is essential for polysaccharides purified from sparassis crispa mediated neuro-protection against glutamate-induced toxicity in differentiated PC12 cells. *International Journal of Molecular Sciences*.

[28] Zhang H., Zhao H., Zhou X. (2016). Isolation and characterization of antioxidant polysaccharides (PKCP-D70-2-a and PKCP-D70-2-b) from the Pinus koraiensis pinecone. *RSC Advances*.

[29] Gu J., Zhang H., Wen C. (2020). Purification, characterization, antioxidant and immunological activity of polysaccharide from *Sagittaria sagittifolia* L. *Food Research International*.

[30] Zhang H., Li J., Xia J., Lin S. (2013). Antioxidant activity and physicochemical properties of an acidic polysaccharide from *Morinda officinalis*. *International Journal of Biological Macromolecules*.

[31] Miao J., Regenstein J. M., Qiu J. (2020). Isolation, structural characterization and bioactivities of polysaccharides and its derivatives from *Auricularia*-a review. *International Journal of Biological Macromolecules*.

[32] Chen J. Y., Sun X. Y., Ouyang J. M. (2020). Modulation of calcium oxalate crystal growth and protection from oxidatively damaged renal epithelial cells of corn silk polysaccharides with different molecular weights. *Oxidative Medicine and Cellular Longevity*.

[33] Huang L. H., Liu H., Chen J. Y. (2020). Seaweed Porphyra yezoensis polysaccharides with different molecular weights inhibit hydroxyapatite damage and osteoblast differentiation of A7R5 cells. *Food & Function*.

[34] Xiang S., Zhou J., Li J. (2015). Antilithic effects of extracts from different polarity fractions of *Desmodium styracifolium* on experimentally induced urolithiasis in rats. *Urolithiasis*.

[35] Ma X.-T., Sun X.-Y., Yu K., Gui B.-S., Gui Q., Ouyang J.-M. (2017). Effect of content of sulfate groups in seaweed polysaccharides on antioxidant activity and repair effect of subcellular organelles in injured HK-2 cells. *Oxidative Medicine and Cellular Longevity*.

[36] Wang K.-P., Wang J., Li Q. (2014). Structural differences and conformational characterization of five bioactive polysaccharides from *Lentinus edodes*. *Food Research International*.

[37] Liu J., Xu L., Gui W., Dorjpalam N., Gerile W. L. (2020). The study on the antioxidant activity of polysaccharides isolated from Polygonatum odoratum (Mill.) Druce. *Mongolian Journal of Chemistry*.

[38] Vardizadeh F., Babaei S., Naseri M., Golmakani M.-T. (2021). Effect of marine sulfated polysaccharides derived from Persian Gulf seaweeds on *Oncorhynchus mykiss* oil stability under accelerated storage conditions. *Algal Research*.

[39] Wang J., Zhang L. (2009). Structure and chain conformation of five water-soluble derivatives of a *β*-d- glucan isolated from *Ganoderma lucidum*. *Carbohydrate Research*.

[40] Pan Y., Wang C., Chen Z., Li W., Yuan G., Chen H. (2017). Physicochemical properties and antidiabetic effects of a polysaccharide from corn silk in high-fat diet and streptozotocin-induced diabetic mice. *Carbohydrate Polymers*.

[41] Luo L., Wu M.-Y., Xu L. (2013). Comparison of physicochemical characteristics and anticoagulant activities of polysaccharides from three sea cucumbers. *Marine Drugs*.

[42] Yang W., Wu J., Liu W. (2021). Structural characterization, antioxidant and hypolipidemic activity of *Grifola frondosa* polysaccharides in novel submerged cultivation. *Food Bioscience*.

[43] Gong L., Zhang H., Niu Y. (2015). A novel alkali extractable polysaccharide from Plantago asiatic L. seeds and its radical-scavenging and bile acid-binding activities. *Journal of Agricultural and Food Chemistry*.

[44] Wang H., Chen J., Ren P., Zhang Y., Onyango S. O. (2021). Ultrasound irradiation alters the spatial structure and improves the antioxidant activity of the yellow tea polysaccharide. *Ultrasonics Sonochemistry*.

[45] Berker K. I., Demirata B., Apak R. (2012). Determination of total antioxidant capacity of lipophilic and hydrophilic antioxidants in the same solution by using ferric-ferricyanide assay. *Food Analytical Methods*.

[46] Machova E., Bystricky P., Malovikova A., Bystricky S. (2014). Preparation and characterization of carboxymethyl derivatives of yeast mannans in aqueous solutions. *Carbohydrate Polymers*.

[47] Yang B. Y., Montgomery R. (2007). Alkaline degradation of invert sugar from molasses. *Bioresource Technology*.

[48] Chiku K., Yoshida M., Ono H., Kitaoka M. (2021). Generation of 3-deoxypentulose by the isomerization and *β*-elimination of 4- *O* -substituted glucose and fructose. *Carbohydrate Research*.

[49] Wang Y. F., Hou G. H., Li J. L., Surhio M. M., Ye M. (2018). Structure characterization, modification through carboxymethylation and sulfation, and *in vitro* antioxidant and hypoglycemic activities of a polysaccharide from Lachnum sp. *Process Biochemistry*.

[50] Guo Q., Xu L., Chen Y. (2019). Structural characterization of corn silk polysaccharides and its effect in H_2_O_2_ induced oxidative damage in L6 skeletal muscle cells. *Carbohydrate Polymers*.

[51] Wang D., Wang D., Yan T. (2019). Nanostructures assembly and the property of polysaccharide extracted from *Tremella Fuciformis* fruiting body. *International Journal of Biological Macromolecules*.

[52] Liu H., Fan H., Zhang J. (2020). Isolation, purification, structural characteristic and antioxidative property of polysaccharides from *A. cepa* L*. var. Agrogatum* Don. *Food Science and Human Wellness*.

[53] Kono H., Kato T. (2021). Elucidation of substituent distribution states for carboxymethyl chitosan by detailed NMR analysis. *Carbohydrate Polymer Technologies and Applications*.

[54] Kono H., Oshima K., Hashimoto H., Shimizu Y. T. K., Tajima K. (2016). NMR characterization of sodium carboxymethyl cellulose: substituent distribution and mole fraction of monomers in the polymer chains. *Carbohydrate Polymers*.

[55] Velichko N. S., Kokoulin M. S., Sigida E. N. (2020). Structural and genetic characterization of the colitose-containing O-specific polysaccharide from the lipopolysaccharide of *Herbaspirillum frisingense* GSF30T. *International Journal of Biological Macromolecules*.

[56] Chakka V. P., Zhou T. (2020). Carboxymethylation of polysaccharides: synthesis and bioactivities. *International Journal of Biological Macromolecules*.

[57] Panat N. A., Maurya D. K., Ghaskadbi S. S., Sandur S. K. (2016). Troxerutin, a plant flavonoid, protects cells against oxidative stress-induced cell death through radical scavenging mechanism. *Food Chemistry*.

[58] Maity P., Nandi A. K., Manna D. K. (2017). Structural characterization and antioxidant activity of a glucan from *Meripilus giganteus*. *Carbohydrate Polymers*.

[59] Patel G. B., Agnew B. J. (1988). Growth and butyric acid production by Clostridium populeti. *Archives of Microbiology*.

[60] Fang Q. H., Zhong J. J. (2002). Submerged fermentation of higher fungus *Ganoderma lucidum* for production of valuable bioactive metabolites --ganoderic acid and polysaccharide. *Biochemical Engineering Journal*.

[61] Albert A., Paul E., Rajakumar S., Saso L. (2020). Oxidative stress and endoplasmic stress in calcium oxalate stone disease: the chicken or the egg?. *Free Radical Research*.

[62] Kwon N., Kim D., Swamy K. M. K., Yoon J. (2021). Metal-coordinated fluorescent and luminescent probes for reactive oxygen species (ROS) and reactive nitrogen species (RNS). *Coordination Chemistry Reviews*.

[63] Tang Z., Zhao L., Yang Z. (2018). Mechanisms of oxidative stress, apoptosis, and autophagy involved in graphene oxide nanomaterial anti-osteosarcoma effect. *International Journal of Nanomedicine*.

[64] Wan B., Wang Z.-X., Lv Q.-Y. (2013). Single-walled carbon nanotubes and graphene oxides induce autophagosome accumulation and lysosome impairment in primarily cultured murine peritoneal macrophages. *Toxicology Letters*.

[65] Zhang C., Wang X., Pi S. (2021). Cadmium and molybdenum co-exposure triggers autophagy via CYP450s/ROS pathway in duck renal tubular epithelial cells. *Science of the Total Environment*.

[66] Zheng R., Zhao L., Chen X. (2021). Metal-coordinated nanomedicine for combined tumor therapy by inducing paraptosis and apoptosis. *Journal of Controlled Release*.

[67] Atale N., Gupta S., Yadav U. C., Rani V. (2014). Cell-death assessment by fluorescent and nonfluorescent cytosolic and nuclear staining techniques. *Journal of Microscopy*.

[68] Wang Z. J., Xie J. H., Nie S. P., Xie M. Y. (2017). Review on cell models to evaluate the potential antioxidant activity of polysaccharides. *Food & Function*.

[69] Chen T., Liu H., Liu J. (2021). Carboxymethylation of polysaccharide isolated from alkaline peroxide mechanical pulping (APMP) waste liquor and its bioactivity. *International Journal of Biological Macromolecules*.

[70] Nuerxiati R., Mutailipu P., Abuduwaili A., Dou J., Aisa H. A., Yili A. (2021). Effects of different chemical modifications on the structure and biological activities of polysaccharides from Orchis chusua D. Don. *Journal of Food Science*.

[71] Alavi Rafiee S., Farhoosh R., Sharif A. (2018). Antioxidant activity of gallic acid as affected by an extra carboxyl group than pyrogallol in various oxidative environments. *European Journal of Lipid Science and Technology*.

[72] Tao Y., Zhang R., Yang W., Liu H., Yang H., Zhao Q. (2015). Carboxymethylated hyperbranched polysaccharide: synthesis, solution properties, and fabrication of hydrogel. *Carbohydrate Polymers*.

[73] Gu Q., Bravo-Diaz C., Romsted L. S. (2013). Using the pseudophase kinetic model to interpret chemical reactivity in ionic emulsions: determining antioxidant partition constants and interfacial rate constants. *Journal of Colloid and Interface Science*.

[74] Yang L., Zhao T., Wei H. (2011). Carboxymethylation of polysaccharides from *Auricularia auricula* and their antioxidant activities *in vitro*. *International Journal of Biological Macromolecules*.

[75] Liu Y. T., You Y. X., Li Y. W. (2017). Characterization of carboxymethylated polysaccharides from *Catathelasma ventricosum* and their antioxidant and antibacterial activities. *Journal of Functional Foods*.

[76] Khan S. R. (2013). Reactive oxygen species as the molecular modulators of calcium oxalate kidney stone formation: evidence from clinical and experimental investigations. *The Journal of Urology*.

[77] Khan S. R. (2014). Reactive oxygen species, inflammation and calcium oxalate nephrolithiasis. *Translational Andrology and Urology*.

[78] Zhu W., Zhao Z., Chou F. J. (2019). The Protective Roles of Estrogen Receptor *β* in Renal Calcium Oxalate Crystal Formation via Reducing the Liver Oxalate Biosynthesis and Renal Oxidative Stress-Mediated Cell Injury. *Oxidative Medicine and Cellular Longevity*.

[79] Konietzny A., Bar J., Mikhaylova M. (2017). Dendritic actin cytoskeleton: structure, functions, and regulations. *Frontiers in Cellular Neuroscience*.

[80] Ordonez D. G., Lee M. K., Feany M. B. (2018). *α*-Synuclein induces mitochondrial dysfunction through spectrin and the actin cytoskeleton. *Neuron*.

[81] Cappiello F., Casciaro B., Mangoni M. L. (2018). A novel *In Vitro* wound healing assay to evaluate cell migration. *Journal of Visualized Experiments*.

[82] De Rechter S., Decuypere J. P., Ivanova E. (2016). Autophagy in renal diseases. *Pediatric Nephrology*.

[83] Liu Y., Li D., He Z. (2018). Inhibition of autophagy-attenuated calcium oxalate crystal-induced renal tubular epithelial cell injury in vivo and in vitro. *Oncotarget*.

[84] Chen Z., Liu X., Ma S. (2016). The roles of mitochondria in autophagic cell death. *Cancer Biotherapy & Radiopharmaceuticals*.

[85] Xu Y., Yang S., Huang J., Ruan S., Zheng Z., Lin J. (2012). TGF-*β*1 induces autophagy and promotes apoptosis in renal tubular epithelial cells. *International Journal of Molecular Medicine*.

[86] Duan Z. H., Duan W. W., Li F. P., Li Y. Q., Luo P., Liu H. Z. (2019). Effect of carboxymethylation on properties of fucoidan from *Laminaria japonica* : antioxidant activity and preservative effect on strawberry during cold storage. *Postharvest Biology and Technology*.

[87] Xu J., Liu W., Yao W., Pang X., Yin D., Gao X. (2009). Carboxymethylation of a polysaccharide extracted from *Ganoderma lucidum* enhances its antioxidant activities *in vitro*. *Carbohydrate Polymers*.

[88] Zou G.-J., Huang W.-B., Sun X.-Y., Tang G.-H., Ouyang J.-M. (2021). Carboxymethylation of corn silk polysaccharide and its inhibition on adhesion of nanocalcium oxalate crystals to damaged renal epithelial cells. *ACS Biomaterials Science & Engineering*.

[89] Wang Z. J., Xie J. H., Shen M. Y. (2016). Carboxymethylation of polysaccharide from *Cyclocarya paliurus* and their characterization and antioxidant properties evaluation. *Carbohydrate Polymers*.

[90] Wang Q., Sheng X., Shi A. (2017). *β*-Glucans: relationships between modification, conformation and functional activities. *Molecules*.

[91] Cai Z., Zhang H. (2021). The effect of carboxymethylation on the macromolecular conformation of the (1 ⟶ 3)-*β* -D-glucan of curdlan in water. *Carbohydrate Polymers*.

[92] Zhang H., Sun X. Y., Chen X. W., Ouyang J. M. (2020). Degraded Porphyra yezoensis polysaccharide protects HK-2 cells and reduces nano-COM crystal toxicity, adhesion and endocytosis. *Journal of Materials Chemistry B*.

[93] Li S., Xiong Q., Lai X. (2016). Molecular modification of polysaccharides and resulting bioactivities. *Comprehensive Reviews in Food Science and Food Safety*.

[94] Luo A., He X., Zhou S., Fan Y., Luo A., Chun Z. (2010). Purification, composition analysis and antioxidant activity of the polysaccharides from *Dendrobium nobile* Lindl. *Carbohydrate Polymers*.

[95] He B., Tao H., Liu S. (2016). Carboxymethylated chitosan protects rat chondrocytes from NO induced apoptosis via inhibition of the p38/MAPK signaling pathway. *Molecular Medicine Reports*.

[96] Wang K., Yang X., Wu Z. (2020). Dendrobium officinale polysaccharide protected CCl4-induced liver fibrosis through intestinal homeostasis and the LPS-TLR4-NF-*κ*B signaling pathway. *Frontiers in Pharmacology*.

[97] Dong N., Li X., Xue C. (2020). Astragalus polysaccharides alleviates LPS-induced inflammation via the NF- *κ*B/MAPK signaling pathway. *Journal of Cellular Physiology*.

